# Evaluation of upper extremity robot-assistances in subacute and chronic stroke subjects

**DOI:** 10.1186/1743-0003-7-52

**Published:** 2010-10-18

**Authors:** Jaka Ziherl, Domen Novak, Andrej Olenšek, Matjaž Mihelj, Marko Munih

**Affiliations:** 1 Laboratory of Robotics, Faculty of Electrical Engineering, University of Ljubljana, Trzaska c. 25, 1001 Ljubljana, Slovenia

## Abstract

**Background:**

Robotic systems are becoming increasingly common in upper extremity stroke rehabilitation. Recent studies have already shown that the use of rehabilitation robots can improve recovery. This paper evaluates the effect of different modes of robot-assistances in a complex virtual environment on the subjects' ability to complete the task as well as on various haptic parameters arising from the human-robot interaction.

**Methods:**

The MIMICS multimodal system that includes the haptic robot HapticMaster and a dynamic virtual environment is used. The goal of the task is to catch a ball that rolls down a sloped table and place it in a basket above the table. Our study examines the influence of catching assistance, pick-and-place movement assistance and grasping assistance on the catching efficiency, placing efficiency and on movement-dependant parameters: mean reaching forces, deviation error, mechanical work and correlation between the grasping force and the load force.

**Results:**

The results with groups of subjects (23 subacute hemiparetic subjects, 10 chronic hemiparetic subjects and 23 control subjects) showed that the assistance raises the catching efficiency and pick-and-place efficiency. The pick-and-place movement assistance greatly limits the movements of the subject and results in decreased work toward the basket. The correlation between the load force and the grasping force exists in a certain phase of the movement. The results also showed that the stroke subjects without assistance and the control subjects performed similarly.

**Conclusions:**

The robot-assistances used in the study were found to be a possible way to raise the catching efficiency and efficiency of the pick-and-place movements in subacute and chronic subjects. The observed movement parameters showed that robot-assistances we used for our virtual task should be improved to maximize physical activity.

## Background

Loss of motor control is a common consequence of stroke [1] and results in many difficulties when performing activities of daily living. Several studies have shown that the use of rehabilitation robotics can improve recovery [2-4]. The benefit of such robots is twofold. First, they can provide objective measurements of the time-course of changes in motor control of the affected limb [5,6]. Second, robots with active motors can be programmed to implement a variety of highly reproducible, repetitive movements and training protocols, allowing patients to semiautonomously practice their movement training [7]. The first device that provided robotic assistance in rehabilitation was the MIT-Manus [8], a 2-degree-of-freedom system that supports planar movements using an impedance controller. The MIT-Manus is augmented with several game-like virtual environments that transform therapy into a fun activity. A more complex device is the MIME [9], which includes several modes of robot-assisted movement: passive, active-assisted and active-constrained. The MIME allows measurement of interaction forces, kinematics, average work per trial and force directional error. Other well-known systems are the ARM Guide [10], which measures and applies assistive or resistive forces to linear reaching movements, and the ADLER [11], which is used to measure the natural wrist movement trajectories seen in real-life functional tasks.

Studies with the aforementioned devices showed that robot-assisted therapy can improve recovery in the long run for both subacute and chronic patients [3,12-15]. Additionally, studies introduced some common measures of performance when using rehabilitation robots as a measuring tool. Casadio et al. [16] estimated the movement duration, linearity of the movement and symmetry of the movement. Harwin et al. [2] listed time to reach a target, the number of velocity peaks, the average or summed interface force with the robot as examples. The study using the MIME robotic device [9] also observed the force in the direction of the movement and average work per trial. If we extend the measures to grasping, the correlation between the grasping and load force has been often employed in research of human motion and grasping [17,18] as a measure of the level of coordination between grasping and movement.

Most of these studies focused on observing the effects of robotic assistance under controlled circumstances. Subjects performed repetitive, predefined arm movements in the robot workspace. Our study includes a complex virtual task: a dynamic environment where movements are subjective and not fully predictable, requiring the subject to be focused and perform considerable physical activity. The aforementioned studies were previously focused on reaching movements and pick-and-place movements while object grasping was not included. The grasping component is also implemented in our virtual task. The rehabilitation outcome of robot-aided therapy compared to classical therapy has already been investigated, so this is not the purpose of this study. The goal of our work was to implement different modes of robot-assistance in a complex virtual environment and evaluate how they affect the subjects' ability to complete the task. We were interested in the impact of various haptic parameters included in the human-robot interaction. Catching efficiency and pick-and-place efficiency are chosen as the indicators of the task performance. Mean reaching forces, deviation error, mechanical work and correlation between the grasping force and the load force are the observed parameters of the human-robot interaction.

## Methods

### MIMICS MMS System specification

The MIMICS multimodal system with the HapticMaster robot (Moog FCS Inc.) was used in the study. This system has already been used in a study where psychophysiological responses were measured and evaluated in stroke subjects [19]. It is an admittance-controlled end-effector-based haptic interface with one rotational and two translational degrees of freedom. A grasping mechanism is attached to a gimbal that allows reorientation of the subject's hand. The mechanism is upgraded with a one degree of freedom finger opening and closing subsystem in order to provide grasping and object carrying capabilities. The hand opening and closing subsystem can be inverted, making the exercise possible for left-and right-handed subjects. Support of the lower and upper arm is provided by an active gravity compensation mechanism. The graphic environment is presented to the subject on a back-projection screen via LCD projector.

### Subjects

Twenty-three subacute hemiparetic subjects (age 51.0 ± 13.3 years, age range 23-69 years, 16 males, 7 females), ten chronic hemiparetic subjects (age 45.6 ± 13.0, age range 30-71 years, 8 males, 2 females) and a control group (twenty-three subjects, age 50.5 ± 12.6 years, age range 24-68 years, 16 males, 7 females) participated in the study. As a result of the stroke, 13 subacute subjects suffered from hemiparesis of the left side of the body and 10 suffered from hemiparesis of the right side. All were right-handed before the stroke. Six chronic subjects suffered from hemiparesis of the left side of the body and 4 suffered from hemiparesis of the right side. They were also all right-handed before the stroke. The stroke subjects were undergoing motor rehabilitation at the University Rehabilitation Institute of the Republic of Slovenia in Ljubljana. The subjects in control group had no physical or cognitive deficits. All were right-handed. To better match the control group and the subacute stroke group, 13 controls performed the tasks with their left hand while 10 performed the tasks with their right hand.

### Experiments

**Figure 1:**
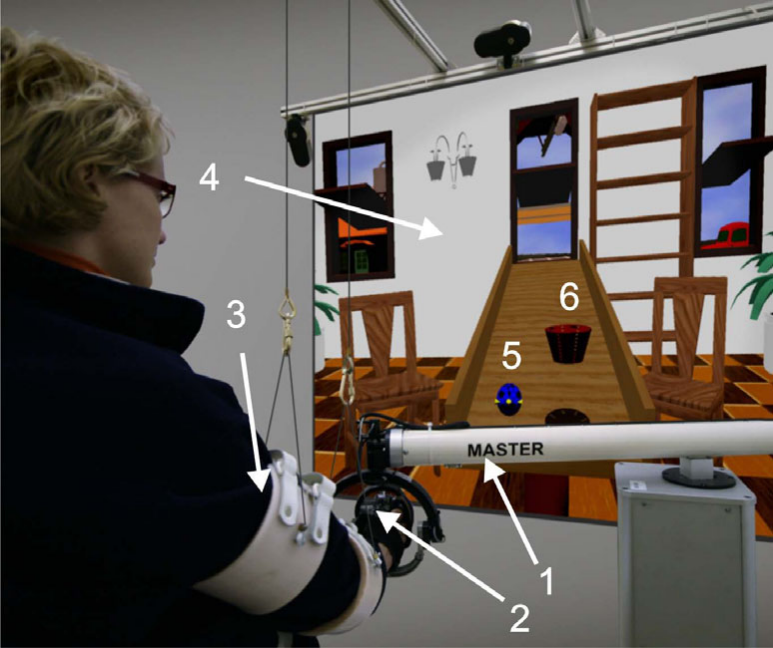
**Rehabilitation system**. A subject performing the virtual rehabilitation task. The subject performs the task using the robot (1) and grasping device (2) while his/her arm is gravity compensated (3). The screen (4) shows an inclined table, a ball (5) and a basket (6).

Before the study began, ethical approval was obtained both from the National Medical Ethics Committee of the Republic of Slovenia and from the Medical Ethics Committee of the University Rehabilitation Institute of the Republic of Slovenia. The rehabilitation task is a catch-and-place exercise. An inclined table is positioned in a room with several objects in the scene (Figure 1). A small sphere and two small cones on the left and right sides of the sphere represent the current position of the robot end-point in the virtual environment. The robot end-point is the point at the top of the robot where the grasping mechanism is attached to the robot. When the subject squeezes the grasping mechanism, the cones move closer together and when the subject releases it, the cones move farther apart. A ball rolls from the opposite side of the table. The subject needs to catch the ball and place it in a basket which appears when the ball is grasped. After the ball is successfully placed in the basket, a new ball rolls down the inclined table. The task is a combination of catching, grasping, pick-and-place movement and releasing.

The task includes different options of robot-assistance. These include:

1. **Catching assistance**. The catching assistance helps the subject to reach the catching point. It is realized by the use of an impedance controller that moves the subject's arm in the frontal plane. The assistance generates the forces when the ball reaches the center of the table, thus giving the subject enough time to reach the catching point by him/herself. The force increases as the ball gets closer to the robot end-effector.

2. **Grasping assistance**. Instead of the manual grasping, the grasping assistance causes the ball to stick to the virtual gripper. When the subject reaches the basket, the ball is dropped automatically. If the grasping assistance is disabled, the grasping force produced by the subject needs to be higher than a reference force. The reference force can be changed during the task according to the subject's grasping ability.

3. **Tunnel assistance**. The haptic trajectory tunnel enables movement from the catching point to the placing point along a predefined trajectory in a virtual haptic environment. An impedance controller prevents the subject from deviating largely from the desired trajectory. The bisector of the tunnel is generated using B-splines and control points. The control points are approximated by using B-splines from trajectories measured in healthy subjects' movements [20]. The guidance assistance provides a force in the direction of the haptic trajectory tunnel. An impedance controller leads the subject's arm along the desired trajectory.

The subjects first tested the virtual rehabilitation environment task for 2 minutes to familiarize themselves with it and find out if they were unable to perform a particular component of the task. They were instructed to try as hard as possible while avoiding extremely tiring or painful activity. The assistances were activated by a therapist based on the testing and stayed the same during the 6-minute training session. Therefore, 7 subacute subjects had grasping assistance, 5 had catching assistance and 7 had tunnel assistance. Seven chronic subjects had grasping assistance, 4 had catching assistance and 5 had tunnel assistance. The control group performed the task without any assistance. Several haptic parameters were measured during training including robot positions, interaction forces between the robot end-point and user, grasping force, position of the ball and a parameter which indicates the task states (the ball is caught, the ball is placed, the ball is missed).

### Evaluation parameters and data analysis

The positions of the robot and the forces were smoothed with a weighted moving average filter (25 weighted samples, all weights equal to 1/25) during the task. The control loop executed at 2500 Hz while the data were sampled at 100 Hz. To analyze performance of the subjects, we observed the following indicators:

1. **Efficiency**. The catching efficiency is the percentage of caught balls divided by the number of all balls. The placing efficiency is the percentage of the balls which were successfully placed in the basket divided by the number of caught balls.

2. **Mean Reaching Forces**. The mean reaching forces at the end-effector sensor can provide information about the direction of the intended movement. These forces were assessed from the time the ball reached the center of the table to the time the ball was caught. The sign of the force is set with respect to the position of the ball. The positive sign represents the force toward the ball, while the negative sign represents the force away from the ball. Only the horizontal component of the force was observed since this component represents the left-right movement of the subject's arm.

3. **Deviation Error**. This is the percentage of the maximal deviation of the measured movement trajectory from a reference line normalized by the reference line length. The reference line is the central line of the tunnel.

4. **Mechanical Work**. The mechanical work is computed from the measured forces at the end-effector and the end-effector positions. The computed work evaluates the interaction between the subject and the haptic robot. Therefore, it is not only the mechanical work performed by the subject. The interaction work toward the target and away from the target were distinguished. The work away from the target represents the resistive work when the guidance assistance is enabled.

5. **Correlation between the grasp force and the load force**. The grasping forces measured during a single pick-and-place movement are divided into three phases: grasping phase, transport phase and release phase. The characteristic point of the grasping phase is when the grasp force reaches the rising time end-point. Rise time is the time required for the grasping force to change from 10% value to 90% value. The characteristic point of the transport phase time is the central point between the grasp and the release. The characteristic point of the release phase is the fall time end-point. Fall time is de fined as the time required for the grasping force to change from 90% value to 10% value. The load force is the vertical component of the end-effector force applied by the subject. Pearson correlation coefficients were computed between the grasping force and the load force for each grasping phase and for each trial. This measure is considered as a sensitive parameter for precision of the coupling between the grasping and load force [18]. A tight coupling is seen in different movements of varying length and direction [21].

For each analyzed parameter, a one-way ANOVA was first used to compare the three groups without assistance (control, stroke, chronic). Then, a two-way ANOVA (assistance × group) was used to evaluate the effect of different modes of haptic assistance (enabled/disabled) on each parameter for both groups (subacute/chronic). Bonferroni corrections were used in post-hoc tests. The control group was not included in the two-way ANOVA since no controls used any kind of haptic assistance.

## Results

### Catching

Comparison of the three groups without catching assistance (controls, subacute, chronic) revealed significant differences in both catching efficiency and mean reaching forces (Table 1). For catching efficiency, post-hoc tests found that the control group caught more balls than the subacute group (p < 0.001) while the difference between control and chronic groups was not significant. For mean reaching forces, controls applied lower forces than both the subacute (p = 0.004) and control (p = 0.003) groups. Two-way ANOVA (catching assistance × group) found a significant main effect of catching assistance on catching efficiency (p = 0.037), with no significant differences between subacute and chronic groups as well as no group-assistance interaction.

**Table 1:** Catching.

	Subacute dCA (n = 18)	Subacute CA (n = 5)	Chronic dCA (n = 6)	Chronic CA (n = 4)	Control dCA (n = 23)
CE [%]	63 ± 17	86 ± 14	62 ± 21	78 ± 27	86 ± 13
MF [N]	0.26 ± 0.26	-0.28 ± 0.51	0.11 ± 0.15	-0.42 ± 0.43	0.03 ± 0.07

The results of observed catching efficiency (CE) and mean forces (MF) during the catching phase of the task. The subacute and chronic subjects are divided into the groups with catching assistance (CA) and without catching assistance (dCA). n is the number of subjects.

### Pick-and-place movements

**Figure 2:**
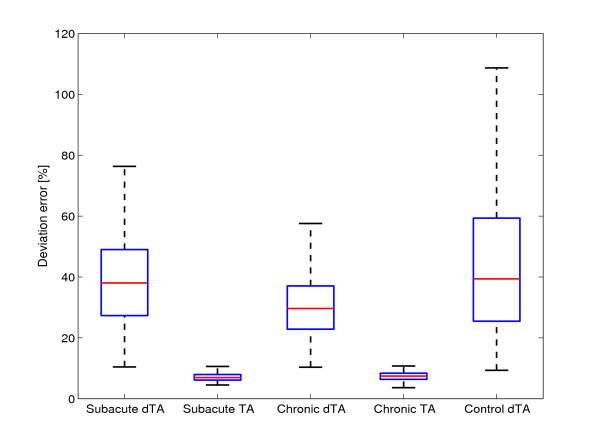
**Deviation error**. Deviation error of the pick-and-place movement with respect to the predefined central curve line. The results are shown for subacute, chronic and control group without tunnel assistance (dTA) as well as for subacute and chronic group with tunnel assistance (TA).

**Figure 3:**
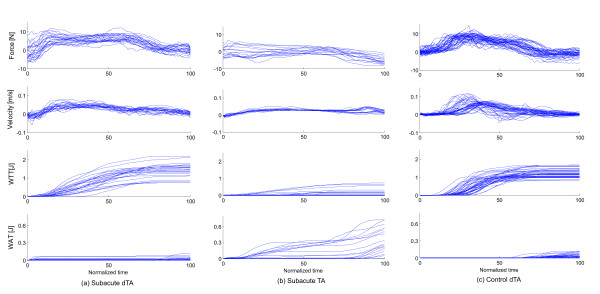
**Measured movement parameters**. Comparison of measured parameters in a subacute dTA subject (a), a subacute TA subject (b) and a control subject (c). The end-effector force, the movement velocity, the work toward target (WTT) and the work away from target (WAT) are shown. The parameters are observed in the tangential direction on the central curve line. The lines represent different trials for the same subject.

**Figure 4:**
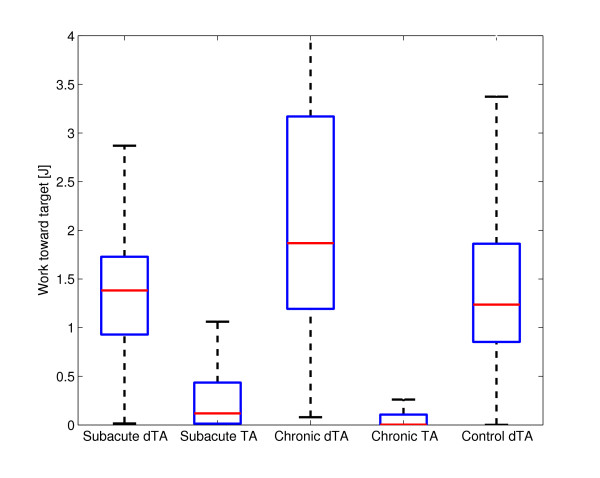
**Work toward the target**. Comparison of the performed work toward the target during pick-and-place movement for the subacute, chronic and control group with disabled tunnel assistance (dTA). The results of chronic and subacute group with tunnel assistance (TA) are also shown.

**Figure 5:**
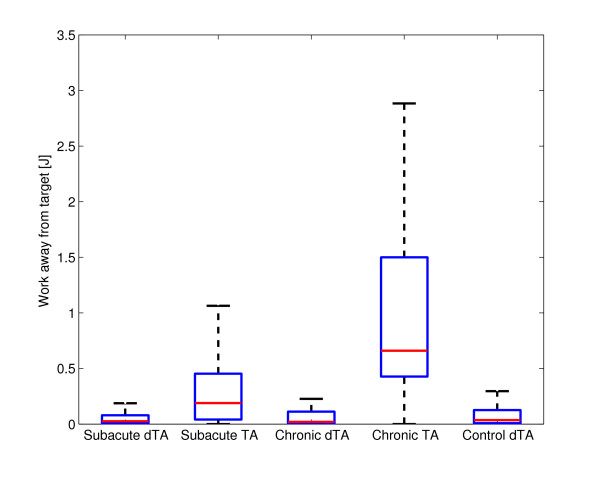
**Work away from the target**. Comparison of the performed work away from the target during pick-and-place movement for the subacute, chronic and control group with disabled tunnel assistance (dTA). The results of chronic and subacute group with tunnel assistance (TA) are also shown.

Comparison of the three groups without tunnel assistance (controls, subacute, chronic) revealed significant differences in pick-and-place efficiency, deviation error and work toward the target (Table 2). Post-hoc tests found that the control group performed pick-and-place movements more successfully than both the subacute and chronic groups (p < 0.001 in both cases). The chronic group had a lower deviation error and performed more work toward the target than both the subacute and control groups (p < 0.001 in all cases). Figure 2 shows the deviation error of the stroke subjects with and without tunnel assistance as well as the deviation error of the control group. The end-effector force, the velocity of the end-effector, the work toward target and the work away from target in the tangential direction of the tunnel are presented in Figure 3. They are shown for one subacute subject without assistance, one subacute subject with tunnel assistance and one control group subject. The time from pick to place point is normalized. Figure 4 shows the work performed toward the target while Figure 5 shows the work performed away from the target for single pick-and-place movements.

**Table 2:** Pick-and-place movement

	Subacute dTA (n = 16)	Subacute TA (n = 7)	Chronic dTA (n = 5)	Chronic TA (n = 5)	Control dTA (n = 23)
PE [%]	79 ± 14	98 ± 6	78 ± 16	100 ± 0	91 ± 9
DE [%]	37.9 ± 16.4	6.9 ± 1.8	29.4 ± 18.2	7.4 ± 3.4	39.4 ± 26.8
WTT [J]	1.39 ± 0.65	0.12 ± 0.38	1.87 ± 1.55	0.01 ± 0.17	1.23 ± 0.91
WAT [J]	0.02 ± 0.40	0.18 ± 0.28	0.19 ± 0.38	0.66 ± 0.83	0.03 ± 0.27

The results of placing efficiency (PE), deviation error (DE), work performed toward the target (WTT) and work performed away from the target (WAT). The subacute and chronic subjects are divided into the groups with tunnel assistance (TA) and without tunnel assistance (dTA). n is the number of subjects.

Two-way ANOVA (tunnel assistance × group) found significant main effects of tunnel assistance on pick-and-place efficiency (p = 0.011), deviation error (p < 0.001), work toward the target (p < 0.001) and work away from the target (p < 0.001). Significant main effects of group (subacute/chronic) were observed on work toward the target (p = 0.003) and work away from the target (p < 0.001). Significant interaction effects were observed on work toward the target (p = 0.003) and work away from the target (p < 0.001).

### Grasping

**Figure 6:**
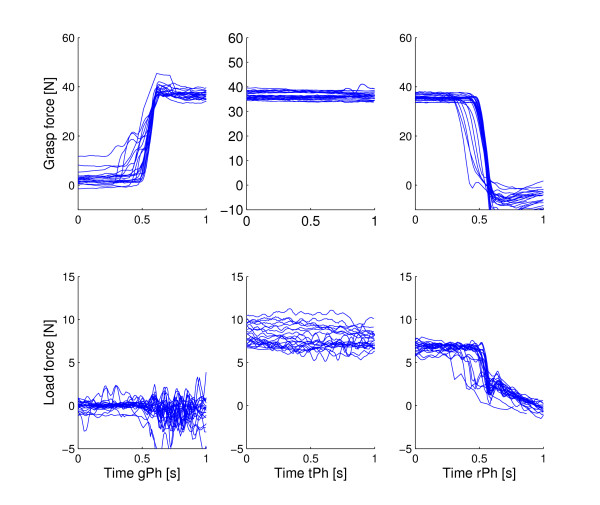
**The grasping force and the load force**. The grasping force and the load force during pick-and-place movement for grasping phase (gPh), transport phase (tPh) and release phase (rPh). The movements were performed by a subacute subject who had no grasping assistance. Each line represents the force during single pick-and-place movement.

**Figure 7:**
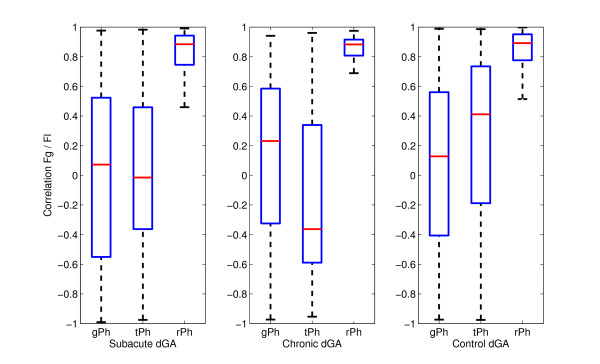
**The correlation between the grasping force and the load force**. Correlation between the grasping force (*F_g _*) and the load force (*F_l_*) for each phase separated: grasping phase (gPh), transport phase (tPh) and release phase (rPh). The load force is the vertical component of the measured force on the end-effector. The results are shown for subacute, chronic, and control group who had no grasping assistance (dGA).

Figure 6 shows the grasping force and the load force during pick-and-place movement in virtual task training for one subacute subject. The forces are observed for grasping, transport and release phase. The Pearson correlation coefficient is computed for each movement in each phase (Table 3). The correlations for the subacute, chronic and control groups are shown in Figure 7. Only the subjects who had grasping assistance disabled are considered. While the correlations are widely spread from -1 to 1 in the grasping and transport phase, the correlation between grasp force and load force exists in release phase. These results are shown for subacute, chronic and control groups. While there are no significant differences among groups in grasping and release phases (p = 0.210; p = 0.218), there is a significant difference between the control group and the other two groups during transport phase (p < 0.001 for both cases). There is a difference in grasping rise time between the subacute and control groups (p = 0.004). The rise time of the grasping force is longer in the chronic group than in control (p < 0.001) or subacute group (p < 0.001). These relationships are similar for the fall time of the grasping force. There are no differences between subacute and control group (p = 0.481) while the chronic group had a longer fall time compared to subacute (p < 0.001) and control (p < 0.001) groups.

**Table 3:** Grasping

	Subacute dGA (n = 16)	Chronic dGA (n = 3)	Control dGA (n = 23)
RT [s]	0.14 ± 0.45	0.47 ± 0.40	0.17 ± 0.34
FT [s]	0.33 ± 0.30	0.54 ± 0.15	0.29 ± 0.39
CGP [-]	0.03 ± 0.58	0.23 ± 0.58	0.12 ± 0.58
CTP [-]	0.01 ± 0.51	-0.36 ± 0.59	0.41 ± 0.58
CRP [-]	0.90 ± 0.40	0.88 ± 0.42	0.89 ± 0.30

Results for grasping force rise time (RT), grasping force fall time (FT), correlation between grasp force and load force for grasping phase (CGP), transport phase (CTP) and release phase (CRP). These groups had grasping assistance disabled (dGA). n is the number of subjects.

## Discussion

As expected, results showed that the stroke subjects had lower catching efficiency than the control group. The subjects reached the same level of efficiency when the catching assistance was applied. Therefore, the catching assistance is a promising tool in certain phase of rehabilitation to raise the efficiency even if it is realized by a simple impedance controller. On the other hand, the mean catching forces showed that the interaction force pointed in the opposite direction when the assistance guided the subject. In most cases, this means that the subjects let the assistance make the movement without making any effort themselves. The reason why control subjects had such low mean forces is that they usually reached the right spot before the ball came into the catching zone. A more complex adaptive assistance model could be the answer to decrease this parameter [22]. Adaptive control algorithms adapt the controller parameters based on measurements of the subjects's performance. Therefore, the assistance is automatically tuned to the subject's individual needs.

The relationships between groups in placing efficiency are similar, except for the subjects with tunnel assistance who had close to 100% efficiency. The deviation error showed that the tunnel greatly limits the movements while the linearity error range in other groups was extended. The control, subacute dTA, and chronic dTA subjects chose the movements that strayed far away from the central line of the tunnel. These findings show that limiting the pick-and-place movement with a haptic tunnel is not the best type of aid at least for this virtual task. In Figure 3, we can see that the peaks of the measured force toward the target are greater in subacute dTA and control subjects than in subacute TA subjects. The positive and negative force of the subacute subjects with the tunnel assistance was in the same proportion while the subjects without the tunnel had mainly positive measured forces. Also, the velocity peaks in the direction toward target are greater in subacute dTA and control subjects than in subacute TA subjects. The velocity profiles are more linear in the subacute subjects who had the tunnel assistance. The tunnel assistance therefore limits the velocity of the pick-and-place movements. The resistive work prevailed the work toward target when the guidance was applied. Therefore, the robot performed most of the movement while the subject was passive. The question remains if the guidance assistance should be applied to the subjects [23,24]. If the subject is not able to perform the movement, the assistance is definitely needed. Other studies showed that adaptive guidance assistance could present a more suitable option [13,23]. However, the haptic tunnel could be an adequate assistance for initial motor learning. The subjects who needed tunnel assistance should train with easier tasks. In our opinion, easier tasks present a better solution than the false feeling of the subject that he or she is able to perform the movement in a more complex task while the robot accomplishes all the necessary work.

The grasping force parameters were examined for the subjects without the grasping assistance. The chronic group had longer rise and fall times than the other two groups. The results showed that the correlation between the load force and the grasping force exists in the release phase. The correlation is not evident in the other two phases. These results are specific for our dynamic task, while other studies showed high correlation along the whole movement [17,18]. Of course, the types of the tasks in these studies were different from ours. This suggests that correlation could be dependant on the task type. Momentary grasping assistance showed no significant changes in the groups that had the assistance, so another type of grasping assistance could be adequate. If we compare all results among the groups, the subacute group without any assistance had comparable results with control group. The chronic group without any assistance deviated more, but the number of subjects in this group is smaller.

## Conclusions

Various clinical studies with robotic devices showed that robot-assisted therapy can improve recovery. Our study was aimed at studying the influence of robotic assistance in a dynamic virtual environment. Rehabilitation robots with their measurement possibilities provide objective performance information. The results of the observed evaluation parameters showed significant differences when different robot-assistive modes were applied to the subjects. Properly applied robot-assistive modes enabled the subject to focus on a particular function of the exercise, such as reaching or grasping, or coordinated actions that combine reaching and grasping. In clinical environments, it is important to appropriately customize the difficulty level in a way to a meet particular patient's performance capabilities. An interesting virtual environment might increase motivation and change the rehabilitation into a fun activity for some subjects as well. In the future, adaptive robot-assistance for pick-and-place movements as well as for grasping assistance will be implemented, to continuously adapt to patient's capabilities during the upper extremity rehabilitation.

## Competing interests

The authors declare that they have no competing interests.

## Authors' contributions

The overall design of the experiments was agreed by all the authors. JZ, AO and MMi developed all related programs and implemented the study. DN carried out the experiments and performed the statistical analysis. JZ and MMu analyzed the data and drafted the manuscript. All authors read and approved the manuscript.
